# A systematic review of psychological, physical health factors, and quality of life in adult asthma

**DOI:** 10.1038/s41533-019-0149-3

**Published:** 2019-10-21

**Authors:** Sabina Stanescu, Sarah E. Kirby, Mike Thomas, Lucy Yardley, Ben Ainsworth

**Affiliations:** 1 Academic Unit of Psychology, University of Southampton, Southampton, UK; 2 NIHR Southampton Respiratory Biomedical Research Unit, University of Southampton, Southampton, UK; 3 Primary Care and Population Sciences, University of Southampton, Southampton, UK; 4 Department of Psychology, University of Bath, Bath, UK

**Keywords:** Asthma, Outcomes research, Quality of life

## Abstract

Asthma is a common non-communicable disease, often characterized by activity limitation, negative effects on social life and relationships, problems with finding and keeping employment, and poor quality of life. The objective of the present study was to conduct a systematic review of the literature investigating the potential factors impacting quality of life (QoL) in asthma. Electronic searches were carried out on: MEDLINE, EMBASE, PsycINFO, the Cochrane Library, and Web of Science (initial search April 2017 and updated in January 2019). All primary research studies including asthma, psychological or physical health factors, and quality of life were included. Narrative synthesis was used to develop themes among findings in included studies in an attempt to identify variables impacting QoL in asthma. The search retrieved 43 eligible studies that were grouped in three themes: psychological factors (including anxiety and depression, other mental health conditions, illness representations, and emotion regulation), physical health factors (including BMI and chronic physical conditions), and multifactorial aspects, including the interplay of health and psychological factors and asthma. These were found to have a substantial impact on QoL in asthma, both directly and indirectly, by affecting self-management, activity levels and other outcomes. Findings suggest a complex and negative effect of health and psychological factors on QoL in asthma. The experience of living with asthma is multifaceted, and future research and intervention development studies should take this into account, as well as the variety of variables interacting and affecting the person.

## Introduction

Over 235 million people worldwide are living with asthma, which is one of the leading non-communicable diseases worldwide.^1,2^ Symptoms, exacerbations, and triggers in asthma are associated with lower quality of life (QoL), tiredness, activity limitation, negative effects on social life and relationships, problems with finding and keeping employment, and reduced productivity.^3–7^ People with asthma are up to six times more likely than the general population to have anxiety or depression,^8^ and 16% of people with asthma in the UK have panic disorder,^9^ compared to 1% in the general population.^10^ People with brittle asthma (difficult-to-control asthma with severe, recurrent attacks) demonstrate even greater comorbidity and maladaptive coping styles.^11^ Psychological dysfunction is often unrecognized in primary care, despite being significantly associated with poor asthma outcomes, including asthma control and QoL.^8,12,13^ Indeed, the European Asthma Research and Innovation Partnership has identified understanding the role of psychological factors as an unmet need in improving asthma outcomes.^14,15^ They propose that anxiety and depression are present at all three stages of the experience of asthma: onset, progression, and exacerbation.^14^

A recent meta-analysis found that asthma diagnoses significantly increased the risk of psychological and health conditions (such as cardiovascular/cerebrovascular diseases, obesity, hypertension, diabetes, psychiatric and neurological comorbidities, gut and urinary conditions, cancer, and respiratory problems other than asthma).^16^ In addition, studies have pointed towards an impact on QoL in people with asthma of additional health and psychological factors, such as comorbid anxiety or depression, higher body mass index(BMI), professional status, and feelings of lack of control over health (for example, refs ^17,18^). Such evidence reinforces the argument that the needs of people with asthma should be approached in conjunction with these additional factors, rather than using a single-illness approach, aiming to reduce the adversity of people’s experience. However, the extent to which psychological and physical health factors interact and impact asthma outcomes is yet to be systematically explored. This systematic review aims to provide a narrative synthesis of the literature exploring psychological and physical health factors that influence QoL in adults with asthma.

## Results

### Study characteristics

**Fig. 1:**
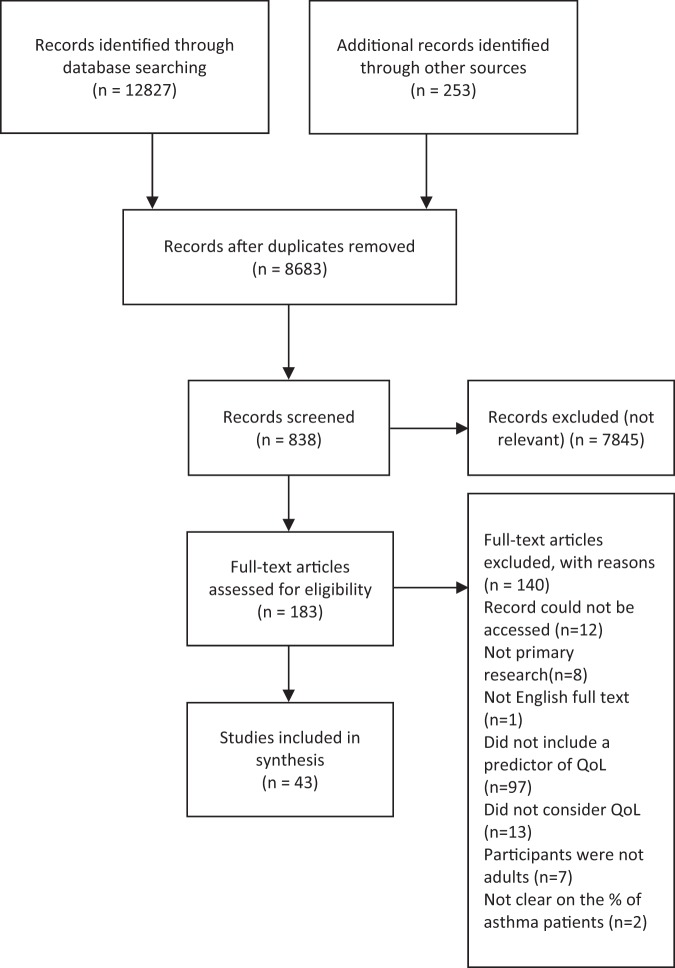
PRISMA statement of included and excluded papers

The search and screening process identified 43 eligible papers, published between 2003 and 2019 (see Fig. 1 for PRISMA flowchart^19^). The characteristics of each study are summarized below in Table 1. Twelve studies were conducted in Europe,^20–31^ 17 in North America,^12,32–47^ 7 in Australia,^17,48–53^ 4 in Asia,^54–57^ and 3 in Africa.^58–60^ All papers employed a quantitative approach comprising 2 longitudinal studies^31,44^ and 41 cross-sectional studies. Only 4 studies included a control group.^21,28,29,31^ Overall, the majority of papers had a large sample size (ranging between 40 and 39,321 participants; 30 papers included a sample size of >100). The majority of studies recruited from primary care or the general population, using self-report to confirm a diagnosis of asthma. Only a few studies recruited from secondary and tertiary asthma clinics.^12,27,36,41,44,48,60^ There was a high occurrence (*n* = 14) of exclusion criteria relating to specific demographic or asthma characteristics, as well as mental health conditions and comorbidities, which restricted the study sample without a reason being given. Most studies used self-report measures,^17,20–30,32–37,39,41–46,48,49,51–60^ with a small proportion using psychiatric interviews to screen for mental health conditions.^12,31,38,40,50^ The majority of studies used asthma-specific QoL measures (*n* = 29),^12,21,23,25,27,28,30,32–37,39–42,44,48–51,54–56,58–61^ 17 included an health-related QoL measure (*n* = 18),^17,20,22–25,28,30,31,34–36,38,43,50–52,55^ and 4 used general measures of QoL (*n* = 7);^26,35,45–47,57,62^ 11 papers used >1 measure of QoL.^23,25,28,30,34–37,50,51,55^ The average age across included studies was 42.1 years (and 61.57% were female). Papers report prevalence rates of between 16.8% and 48.9% for depression and between 13.3% and 44.4% for anxiety,^20,27,33,38,50,56,58,60^ with an average of 28.31% for a diagnosis of anxiety or depression. Across several studies, the prevalence of other mental health conditions was 28.31% on average (ranging between 28% and 80%).^12,37,38,40,42^ Between 72% and 86.9% of people with asthma had at least one additional physical condition and between 21% and 26.3% had ≥2;^25,34,56^ 26.36% had, on average, at least one other physical health condition. On average, people with asthma were significantly more likely to have a BMI of >30 (and between 61% and 75.1% had a BMI >25).^26,45,59^ The quality assessment identified that most studies were of a reasonable quality; however, it should be noted that some measures used could be considered inappropriate for the research aim or the population under investigation. Examples include measuring general QoL with an asthma-specific measure or administering a geriatric depression questionnaire to a young adult population.

**Table 1:** Characteristics of included studies

Study	Sample	Study design and recruitment	Predictor	QoL measurement	Findings—summary
Adams et al.^17^	7619 people from the general population (834 with asthma)	Cross-sectional, population household interview	Kessler Psychological Distress Scale (K10), for a global measure of psychological distress, containing measures of depressive and anxiety symptoms experienced over 4 weeks+self-report of diagnosed psychiatric conditions	SF-12	Psychological distress was more common in people with asthma (17.9% vs 12.2%, *p* < 0.01); mental health conditions were more common (16.2% vs 12.2%, *p* < 0.01) People with asthma and psychological distress had significantly lower QoL than those with either asthma or psychological distress alone (the physical component summary of the SF-12). Among those with psychological distress, the mental component summary did not differ between people with or without asthma
Adams et al.^51^	293 adults with asthma, at baseline and 232 at 12 months	Longitudinal study (measures at baseline and 12-month follow-up), patients recruited from outpatient clinics, emergency departments and inpatients at 2 hospitals	Coping scales to measure active, avoidance, and denial coping, as well as other measures such as—self-efficacy in asthma, perceived emotional and social support, satisfaction with illness scale	SF-36 and the Modified Marks AQLQ	Avoidance coping and clinical asthma status were significant predictors of the Marks AQLQ and the physical and mental components of the SF-36 in a regression model. Less avoidance was associated in an increase greater than one standard deviation for all scales. Similar trends were observed for active coping and self-efficacy but not denial. Active coping was a significant predictor of the physical component (*r*^2^ = 0.69) and satisfaction with illness was a significant predictor of the mental component (*r*^2^ = 0.54)
Adams et al.^52^	7619 people from the general population (834 with asthma)	Cross-sectional, population household interview	Any additional condition from: diabetes, arthritis, heart disease, stroke, cancer, osteoporosis	SF-12	People with asthma were more likely to report a physical comorbidity (odds ratio 19.9, 95% CI 1.5–2.2) People with asthma and other conditions reported more days unable to do usual activities (16.0 compared to 11.3 with asthma alone and 9.2 with other conditions) When controlling for age and gender, additionally, PCS scores significantly (statistic and clinical) decreased with the presence of an additional condition. Having two or more conditions (one of which was asthma) was associated with a lower SF-12 score than expected from the effects of asthma and the chronic condition alone
Adeyeye et al.^60^	201 adult participants with moderate and severe asthma	Cross-sectional, recruited from an asthma outpatient clinic	The Mini International Neuropsychiatric Interview (M.I.N.I) to assess the presence of anxiety and/or depression	Mini-AQLQ	Presence of anxiety/depression was a significant independent predictor of the mini-AQLQ score and of the emotional domain (*p* < 0.001)
Afari et al.^38^	50 adult participants with confirmed asthma	Cross-sectional, recruited from an asthma clinic	Diagnostic Interview Schedule for DSM-III-R	SF-36	Asthma patients with a lifetime diagnosis of depression reported worse physical functioning, mental health functioning, and health perceptions (*F*s ranged between 2.60 and 4.18, *p*s <0.05). Scores for anxiety followed similar trends but were non-significant
Al-kalemji et al.^62^	778 adult participants completed questionnaires (181 with asthma, 597 without)	Cross-sectional, recruited from an earlier cohort	BMI, 2 questions on the 15D and participants were asked (yes/no) if they had anxiety or depression	15D (measure of global QoL)	Depression was significantly associated with worse QoL on all domains (*r* = –0.076, CI –0.091 to –0.061), but it did not inflate the relationship between asthma severity and QoL (severity had an independent influence on QoL regardless of psychological state) Anxiety (*r* = –0.079, CI –0.101 to –0.056) and obese BMI (*r* = –0.021, CI –0.034 to –0.008) were both significant predictors of QoL
Avallone et al.^39^	127 adult patients with asthma	Cross-sectional, recruited from a community allergy and asthma office	Comorbid conditions: arthritis/rheumatism, frequent or severe headaches, seasonal allergies, heart attack, high blood pressure, diabetes, HIV/AIDS, ulcers, back or neck problems, chronic pain, stroke, heart disease, chronic lung disease, and cancer—the number of conditions was included as a covariate in the analysis; the positive and negative affect schedule (PANAS)—a mood measure to assess negative affect; the anxiety sensitivity for fear of negative consequences from anxiety symptoms	Mini-AQLQ	The number of comorbid conditions was significantly associated with QoL on all domains (range −0.21 to −0.33). Negative affect was associated with all dimensions, except for the environmental stimuli domain. AS-Physical concerns was associated with all QoL domains. A model of gender, age, negative affect and number of medical problems significantly predicted QoL, explaining 20.2% of the variance in symptom-related QoL and 22.7% of the variance in activity limitation (gender and age were not significant independent predictors, but both negative affect and number of medical problems were)
Bohmer et al.^20^	196 participants with a main diagnosis of asthma	Cross-sectional, recruited for a different study from primary and specialist practices	HADS	SF-12	Scores for both anxiety and depression were significantly associated with worse QoL on the physical and mental dimensions. Increasing age, female gender, higher number of medications, and symptoms of depression explained 48% of the variance in the physical component. Living alone and reporting symptoms of anxiety explained 33% of the variance in the mental component
Choi et al.^56^	202 patients: 127 non-elderly (20–64 years) and 75 elderly (>65 years) patients with asthma	Cross-sectional, recruited from five allergy and asthma clinics	Korean version of the PHQ-9	Asthma-Specific QOL (AQOL)	AQOL scores were significantly lower for people with depression and asthma (72.4 vs 98.6, *p* < 0.01); this was true for both groups (elderly and non-elderly) Within the elderly group, a higher BMI was significantly associated with depression. Comorbidities (yes/no) were not more or less prevalent in people with or without depression
Coban and Aydemir^27^	174 adults with asthma	Cross-sectional, consecutive patients recruited from secondary care	HADs and allergic status	AQLQ	There was no difference between people who had atopic and non-atopic asthma in terms of anxiety, depression, or QoL. Participants with a higher general anxiety and depression score had lower QoL (3.62 vs 4.68, *p* < 0.01 for anxiety and 3.81 vs 4.80, *p* < 0.01 for depression). Average scores for people with asthma and anxiety and/or depression were worse than one standard deviation when compared with people with asthma but without anxiety/depression
Deshmukh et al.^61^	110 adult patients with asthma	Cross-sectional, recruited patients who visited an emergency department in the past 18 months	HADs	AQLQ	Anxiety (*R*^2^ = 0.128) was a significant predictor of AQLQ. Having anxiety was correlated with having depression (*F* = 27.17, *p* < 0.001). People with anxiety and depression had significantly lower QoL scores (*F* = 11.54 for anxiety and *F* = 26.3 for depression *p* < 0.001). Overall, symptoms of anxiety and depression accounted for 28.3%; anxiety was significantly correlated with emotional functioning and response to environmental stimuli (subscales of the AQLQ) when controlling for depression
Ekici et al.^28^	116 adult asthma patients and 116 matched healthy controls	Cross-sectional, recruited from a respiratory disease clinic (matched controls recruited from the community of visitors to the same hospital)	Negative mood was evaluated with a questionnaire including six mood subscales in 3 categories—nervous–anxious, hostile–angry, and fearful–panicky	SF-36 and AQLQ	Negative mood scores were not different between people with or without asthma Both components of the SF-36 (mental and physical) were worse in people with asthma. They were associated with negative mood scores (*β* = −0.37 and *β* = −0.28, *p*s <0.01, respectively) Negative mood accounted for 67% of the variance in AQLQ (the impact of negative mood on symptoms and activity domains of the AQLQ was significant but not the emotional and environmental domains)
Erickson et al.^34^	603 adults with asthma	Cross-sectional, recruited patients who were enrolled in a managed care organization	Number of comorbidities and health belief questionnaires (based on the Health Belief Model)	AQLQ and SF-36	Number of comorbidities was significantly associated with decreased QoL on all 10 components and the overall score (*β* ranging from −0.062 to −0.360, significant for summary AQLQ, activity limitation, symptoms and exposure to environmental stimuli, and all components of the SF-36, including composite mental and physical summaries) illness perceptions (symptom-derived severity and perceived severity) were both significant predictors of the physical component of the SF-36 and of all subscales of the AQLQ (*β* values ranged from −0.155 to −0.237)
Favreau et al.^44^	643 adults with asthma	Longitudinal, 4.3 year follow-up, recruited from tertiary care	Primary care evaluation of mental disorders interview (to assess panic disorder), anxiety sensitivity index (to assess panic-anxiety)	AQLQ	Having a diagnosis of PD did not significantly predict total AQLQ scores. Higher anxiety sensitivity at baseline predicted worse symptoms (*β* = −0.013, *p* < 0.01) and emotional distress (*β* = −0.014, *p* < 0.01) but not overall AQLQ. This stayed true when controlling for covariates (age, gender, years of education, smoking, major depression, medication use, and baseline scores)
Faye et al.^57^	60 adults with asthma	Cross-sectional, consecutive patients recruited from an outpatient tertiary care respiratory hospital	DSM-IV-TR criteria for Panic and Agoraphobia (including the Panic and Agoraphobia scale to assess the severity of panic disorder), number of comorbidities	WHO QOL BREF scale and the WHO disability schedule II	83.3% of people with ≥4 panic symptoms (not qualifying for panic attack diagnosis) reported ‘sensations of shortness of breath’, ‘fear of choking’ and ‘fear of dying’ QoL scores were significantly lower on the physical (44.3 vs 49.3) and environmental (43.5 vs 47.6) domains for participants with panic disorder compared to those without panic disorder. All participants with PD had poor QoL (significantly lower when compared to those without)
Goldney et al.^50^	3010 interviews conducted (299 with adults with asthma)	Cross-sectional, population interview (random sample)	Dyspnoea dimension of the AQLQ to determine dyspnoea; PRIME-MD (psychiatric interview tool) to determine depression	AQLQ and SF-36	Increases in major depression were associated with dyspnoea (44.2% with depression and dyspnoea, compared with 17.9% with depression and no dyspnoea, *p* < 0.01) This group of people showed significantly lower scores on all domains of the SF-36 (suggesting that depression could be a mediating factor)
Gonzalez-Barcala et al.^26^	2125 adult participants with asthma	Multi-stage cross-sectional, recruited from primary care clinics	BMI, incidence of stressful events, presence of allergy sensitization	EQ-5D	32% of people with asthma reported ‘serious problems’ on the anxiety/depression scale of the EQ-5D. Stressful events of giving little importance to adherence to treatment were significant predictors of EQ-5D. Having a BMI of <25 was significantly associated with better mobility (OR = 2.14), less activity limitation (OR = 1.43), and less pain (OR = 1.75)
Hommel et al.^32^	64 adolescents and young adults with asthma (aged 18–25 years)	Cross-sectional, recruited from the community	IDD (to assess depression), the Beck Anxiety Inventory, and subjective illness severity	LVAQ	LVAQ was significantly correlated with subjective severity (*r* = 0.48, *p* < 0.01). The combined influence of anxiety and depression accounted for 14% of the variance in QoL; depression contributed significantly to variance in QoL (*t* = 2.65; *p* < 0.05) before anxiety was introduced in the model; anxiety demonstrated a significant main effect on asthma-specific QoL (*t* = 2.58; *p* < 0.05)
Hullmann et al.^43^	74 adult participants with asthma (and 74 with allergies)	Cross-sectional, recruited from a university	Mishel Uncertainty in Illness Scale—to assess 4 components of illness uncertainty (ambiguity, uncertainty, lack of information, and unpredictability); Illness Intrusiveness Scale—to assess the illness-induced interference with various life activities	SF-36	The overall model (including illness uncertainty and illness intrusiveness, gender, and asthma severity) accounted for 59.3% of the variance in SF-36 scores for the physical component and 19.6% for the mental component. Illness intrusiveness and illness uncertainty were significant independent predictors of the physical component but not of the mental component
Kolawole et al.^58^	81 adult patients with asthma	Cross-sectional, consecutive patients recruited from an asthma clinic	HADs	Mini-AQLQ	Presence of anxiety symptoms (*χ*^2^ = 7.9, *p* < 0.05) and depressive (χ^2^ = 6.45, *p* < 0.05) symptoms (according to HADs) was significantly associated with decreased QoL.
Krauskopf et al.^33^	317 participants with asthma aged over 60	Cross-sectional, recruited from outpatient health clinics (secondary care)	PHQ-9 (to assess symptoms of depression)	Mini-AQLQ	Patients with depression showed poorer quality of life than those without (mean score difference in AQLQ = −1.4, *p* < 0.001).
Kullowatz et al.^30^	88 adult patients with asthma	Cross-sectional, recruited from a larger study conducted at a pulmonary clinic	HADs	Living with asthma questionnaire (LVAQ) and SF-12	After controlling for demographics and symptom severity, anxiety accounted for considerable variance in SF-12 mental wellbeing and LAQ psychological wellbeing (explaining 22% and 9% of the variance, respectively). Including depression accounted for additional variance an additional 8% and 2%, respectively For physical wellbeing, depression was significantly associated, explaining 6% of the variance, but not anxiety Significant associations were found between anxiety and depression and the functional subscale of the LAQ (explaining 4% and 3% of the variance, respectively)
Lavoie et al.^12^	406 adult patients with asthma	Cross-sectional, consecutive patients recruited from an asthma clinic	Structured Psychiatric interview—the Primary Care Evaluation of Mental Disorders to detect the most common psychiatric disorders, according to DSM-IV	AQLQ	Despite no differences in pulmonary functions, people with psychiatric disorders reported significantly lower AQLQ on all individual scores and total score (mean score 5.3 vs 4.6, *p* < 0.01)
Lavoie et al.^40^	504 adult patients with asthma	Cross-sectional, consecutive patients with asthma recruited in primary care	Primary Care Evaluation of Mental Disorders—PRIME-MD	AQLQ	Independent effects of depression on AQLQ (*F* = 38.5, *p* < 0.01) and anxiety on AQLQ (*F* = 18.06, *p* < 0.01, total score) but no significant interaction effect (the multiple regression model containing severity, sex, age, depression, and anxiety and the interaction accounted for 22% in the interaction). There was a significant independent effect of depression (explaining 3% of the variance in AQLQ) and anxiety (explaining 1% of the variance). They were significant predictors on every subscale, explaining between 1% and 3% of the variance in AQLQ subscales
Lavoie et al.^42^	557 adults with asthma	Cross-sectional, patients recruited from a larger study conducted in tertiary care.	Psychiatric Interview to assess mental disorders, Asthma Self-Efficacy Scale	AQLQ	ASES scores were significantly correlated with AQLQ, suggesting that being confident in one’s ability to control asthma symptoms is associated with better quality of life (*r* = 0.62, *p* < 0.01). Lower ASES scores were also significantly correlated with a higher BMI and having a comorbid mood or anxiety disorder
Lomper et al.^22^	96 adult patients (33 with controlled asthma, 63 with uncontrolled asthma)	Cross-sectional, recruited from an outpatient allergy clinic	HADs (measured both anxiety and depression but only performed an analysis of correlations between depression and QoL	SF-36	There was a significant difference in the mental component between people with or without depression (51.4 vs 71.8, *p* < 0.05) in the group of people with controlled asthma. There was no significant difference between people with or without anxiety In the uncontrolled asthma group, depression was associated with poorer QoL on both physical and mental components (48.6 vs 30.3 and 57.5 vs 33.7, respectively, *p*s <0.01). Anxiety was also associated with poorer QoL on both physical and mental components (54.8 vs 30.8 and 62.7 vs 40.5, *p*s <0.01)
Maalej et al.^59^	200 adult participants with asthma	Cross-sectional, recruited from outpatient respiratory departments	BMI and presence of comorbidities (out of diabetes, hypertension, hypercholesterolemia, rhinitis, and sinusitis)	AQVAT (Arabic version of the AQLQ)	Higher BMI was correlated with higher number of comorbidities (*p* < 0.01 for diabetes, hypertension, hypercholesterolemia, GERD, rhinitis, and sinusitis) and with lower QoL (11.48 vs 64.35, *p* < 0.01 between people with normal and obese BMI)
Mancuso et al.^36^	230 adult people with asthma	Cross-sectional, recruited from outpatients tertiary care	A screening question for depression and the Geriatric Depression Scale	AQLQ and SF-36	Participants with positive screening scores for depressive symptoms had significantly lower AQLQ and SF-36 scores (as well as significantly worse scores on each individual domain, *p* < 0.05). Depression score was a significant predictor of AQLQ, explaining 23% of the variance
McCormick et al.^41^	44 adults with asthma	Cross-sectional, recruited from secondary care	Maladaptive coping (based on the transactional stress models of health) assessed with the Social Problem Solving Inventory Revised: Short Form	Mini-AQLQ	Controlling for variance associated with gender, age, and income, people with higher impulsive-careless scores scored lower on QoL (*β* = 0.79, *p* < 0.01). Problem-solving style was the only significant independent predictor of QoL
Miedinger et al.^37^	60 adult participants with occupational asthma	Cross-sectional, people recruited after being evaluated for a permanent disability indemnity	Primary Care Evaluation of Mental Disorders—PRIME-MD; Psychiatric Symptoms Index	AQLQ & the St-Georges Respiratory Questionnaire	Significant medium-to-high correlations between the PSI and AQLQ (*r* = −0.619); having any mood or psychiatric disorder according to PRIME-MD showed significant medium correlations with all subscales of the AQLQ (*r* = 0.417 for any psychiatric disorder and composite score of AQLQ)
Nishimura et al.^55^	162 adult patients with mild-to-severe well-controlled asthma	Cross-sectional, consecutive patients recruited from an outpatient secondary care clinic	HADs and presence of dyspnoea	Living with asthma questionnaire (LVAQ) and SF-36	Having anxiety or depression according to HADs scores showed mild but significant correlations with both QoL questionnaires (scores ranging from 0.31 to 0.60). Severity of dyspnoea was also associated with both, with correlation scores ranging from 0.22 to 0.56
Oga et al.^54^	87 adult Patients with stable asthma	Longitudinal, recruited from an outpatient secondary care asthma clinic 6 months after treatment and follow-up 5 years	HADs	AQLQ	Changes in HADs scores were significantly correlated with changes in AQLQ on both anxiety and depression scales (*r* = −0.6, *p* < 0.01 and *r* = −0.5, *p* < 0.01 respectively), but not changes in physiological measures. HADs scores overall remained similar over the 5-year follow-up period
Oguzturk et al.^21^	70 patients (with stable asthma and aged >60 years) and 40 age-matched controls	Cross-sectional, recruited from a secondary care respiratory clinic (matched controls were recruited from local mosques)	HADs	AQLQ	Patients with earlier-onset asthma (duration >8 years) had lower QoL scores than those with recent-onset asthma. Anxiety and depression were significant predictors of AQLQ scores, anxiety accounted for 49% and depression for 41% of the total score
Pate et al.^46^	18,856 people with asthma	Cross-sectional, sample recruited from wider telephone population study of 39,321 (BRFFS sample)	Additional chronic conditions, BMI, presence of depression	General Health, Activity Limitation, Physical/Mental Health Impairment (Yes/No Questions)	Having additional conditions (PR = 4.26), depression (PR = 1.97), as well as either underweight (PR = 1.82), overweight (PR = 1.19), or obese (PR = 1.76) BMI were all significantly associated with ≥14 days of activity limitation, as well as self-rated fair/poor health
Powell et al.^49^	218 pregnant women with asthma and rhinitis	Cross-sectional, recruited from an ante-natal clinic	Rhinitis was assessed using a visual analogue scale, Six Item Short-Form State Trait Anxiety Inventory	AQLQ-M	QoL scores were predicted by the presence of rhinitis, anxiety, and prior history of rhinitis (medians 0.63 vs 1.06, *p* < 0.01 for pregnant women with asthma, with and without current rhinitis)
Sandez et al.^24^	40 adult patients with near-fatal asthma	Cross-sectional, recruited from an outpatient asthma clinic (secondary care)	Beck’s Depression Inventory and the Panic-Fear Scale of the Asthma Symptom Checklist	SF-36 (MCS and PCS components)	Panic-Fear (PF) and age accounted for 22.8% of variance in PCS and depressive symptoms accounted for 48.6% of the variance in MCS. PF was significantly and negatively correlated with both MCS and PCS (*r* = −0.53 and *r* = −0.36, respectively, *p*s <0.05). Depressive symptoms were only significantly correlated with MCS (*r* = −0.69, *p* < 0.05)
Strine et al.^45^	18,856 people with asthma	Cross-sectional, sample recruited from wider telephone population study of 39,321 (BRFFS sample)	PHQ-8, self-report diagnosis of depression, BMI	General Health, Activity Limitation, Physical/Mental Health Impairment (Yes/No Questions)	Among adults with asthma, people with current depression were significantly more likely than those without depression to report more mean numbers of days in the past 30 days of physical distress (OR = 4.7), mental distress (OR = 14.3), activity limitations (OR = 7.0), depressive symptoms (OR = 23.6), anxiety symptoms (OR = 9.8), insufficient sleep (OR = 6.3), pain (OR = 6.0), and fatigue (OR = 13.3). There was a dose response relationship between depression severity and the mean number of days of physical distress, mental distress, depressive symptoms, fatigue, anxiety symptoms, and activity limitations. Those with current depression were also significantly more likely to have an obese BMI
Tay et al.^48^	90 adult patients with difficult asthma	Cross-sectional, consecutive patients recruited from a difficult asthma clinic	Having one of the eight comorbidities: allergic rhinitis, chronic rhinosinusitis, gastro-oesophageal reflux disease, obesity, obstructive sleep apnoea, anxiety or depression, dysfunctional breathing, and vocal cord dysfunction	AQLQ	BMI was an independent predictor of poor QoL (*β* = −0.05, *p* < 0.01). Dysfunctional breathing predicted poor QoL (*β* = −0.73, *p* < 0.05), as did vocal cord dysfunction (*β* = −0.78, *p* < 0.05). On univariate analysis, BMI, VCD, DB, OSA, and GORD were significantly associated with decreased QoL
Urbstonaitis et al.^47^	5857 late midlife adults with asthma	Cross-sectional, sample recruited from wider telephone population study of 39,321 (BRFFS sample)	BMI, presence of respiratory comorbidity	General Health, Activity Limitation, Physical/Mental Health Impairment (Yes/No Questions)	Respiratory comorbidity was significantly associated with poor QoL on all dimensions and independent of asthma control (OR = 17). People with poorly controlled asthma were more likely to have an obese BMI. The combination of poor control and obese BMI was significantly associated with poorer general health (OR = 2.3)
Vasquez et al.^23^	76 adults with asthma	Cross-sectional, recruited from a secondary care pneumology department.	Cognitive Depression Index (subscale of the Beck Depression Inventory); Trait Subscale of the State-Trait Anxiety Scale; the Twenty-Item Toronto Alexithymia Scale—this has three dimensions: DIF, DDF, and EOT	SF-36 and The St George's Respiratory Questionnaire to measure disease-specific impairment	Trait anxiety, depression scores, and alexithymia were included in a regression model that explained between 23% and 39% of variance in QoL. Depression was a significant independent predictor and associated with all subscales of the SF-36, as well as all the subscales of the SGRQ
Vortmann and Eisner^35^	843 adult patients with severe asthma	Cross-sectional, recruited patients who were hospitalized for asthma in the previous 4 years.	BMI from self-reported height and weight, atopic history; Center for Epidemiologic Studies Depression Scale	Marks Asthma QoL Questionnaire and the SF-12 and daily activity restriction	Compared to normal BMI, general physical health was significantly worse in those with obese BMI (mean score decrement of −6.31) and overweight BMI (mean score decrement −2.42). Asthma-specific quality of life was significantly worse in the underweight group (mean score difference 8.66 points) and obese group (4.51 points). People with obese BMI also had a higher number of restricted activity days (5.05 days more). Obese patients had significantly higher risk of depressive symptoms. Depression was found to be a significant mediator of the relationship between obesity and health status, asthma QoL, and restricted activity days
Wijnhoven et al.^25^	395 patients with asthma, aged 40–75	Cross-sectional, participants recruited from general practice	Presence or absence of: diabetes mellitus, hypertension, cardiac disease, cerebrovascular disease, musculoskeletal disease, and malignancies and asked if they had any other chronic condition. Comorbidity was defined as (1) the presence of comorbidity; (2) number of comorbid conditions; (3) presence of specific comorbidity	Disease-specific instrument: Quality of Life in Respiratory Illness Questionnaire; generic instrument: the Dutch version of the Nottingham Health Profile (NHP)	Having one or more comorbidities was significantly associated with poorer asthma-specific QoL (OR = 2.08) and poorer general QoL (OR = 2.96). Poorest QoL was found in patients with more than one comorbid condition (OR = 4.77). Cardiac disease and hypertension were significantly associated with poor disease-specific QoL in asthma, and musculoskeletal disorders were most strongly associated with poor general QoL
Yilmaz et al.^31^	97 adult patients with asthma and 97 healthy controls	Cross-sectional, recruited from a secondary care outpatient chest disease clinic	SCID-II (structured method of interview, according to the DSM-III-R to diagnose axis II personality disorders)	SF-36	People with asthma and personality disorders had significantly lower QoL scores than people with asthma and no personality disorders. This was significant for physical role functioning (42.68 vs 62.50, *p* < 0.05), general health (38.56 vs 53.60, *p* < 0.01), and mental health (53.75 vs 65.55, *p* < 0.01). All physiological measures (FEV, severity of asthma, disease duration, etc.) were not significantly different between people with or without personality disorders

### Narrative synthesis

Narrative synthesis generated three overarching themes: psychological factors, health factors, and multifactorial aspects (see Table 2 for themes and subtheme descriptions). Overall, patients with asthma demonstrated impaired QoL, which was further decreased by psychological factors (e.g. anxiety, depression, emotion regulation, illness perceptions), health risk factors (such as an increased BMI), and the presence of a co-existing mental health or physical condition (such as rhinitis, cardiovascular disease, diabetes, etc.). Having more than one co-existing condition or psychological factor impacted overall QoL even more substantially. Results for each of the aspects found are presented below.

**Table 2:** Themes, subthemes, and descriptions

Theme	Subtheme	Description
Psychological factors	Anxiety and depression	Included people with clinical anxiety or depression,^12,17,27,32,33,38,40,42,44–46,48,50,56,57,60–62^ as well as people showing symptoms (or scoring high on scales, such as the HADS)^17,20–24,27,30,36,37,54,55,58,61,62^
	Other mental health conditions	Panic disorder with or without agoraphobia,^24,38,44,57^ personality disorders,^31^ alexithymia,^23^ somatization,^38^ mood disorders,^12,40,57^ schizophrenia, eating disorders, substance use disorders,^38^ and general occurrence of any psychiatric disorder^12,17^
	Emotion regulation	Negative affect^28,39^ or coping^41,51^
	Illness representations	Illness-related cognitions,^26,34,37,42,43,51,60^ subjective illness severity, uncertainty in illness, illness intrusiveness,^43^ perceived disability,^60^ health beliefs and attitudes, perceived severity,^34^ self-efficacy, satisfaction with illness,^51^ anxiety sensitivity to physical concerns,^39^ and satisfaction with life^37^
Physical health factors	Physical health conditions	Diabetes,^25,48^ obesity,^48^ hypertension,^25,39^ gastro-oesophageal reflux disorder,^48^ rhinitis,^48,49^ vocal cord dysfunction,^48^ sleep apnoea,^48^ musculoskeletal disorders,^25,39^ arthritis,^39,52^ heart disease,^25^ stroke,^39,52^ cancer,^39,52^ osteoporosis,^52^ dysfunctional breathing,^48^ headaches^39^ and allergic status,^27,39^ or the presence of additional chronic conditions^25,27,33,34,36,39,46–49,52,53,56,59^
	BMI	BMI^25,26,28,29,35,42,44–48,56,59^
Multifactorial aspects		Interactions between conditions, BMI, psychological factors, and anxiety and depression^17,35,42,45,50,56,59^

### Psychological factors

Within this first theme, four subthemes were generated. These comprised ‘anxiety and depression’, ‘other mental health conditions’, ‘emotional regulation’, and ‘illness representations’.

Anxiety and depression were notably the most commonly considered factors (*n* = 30). A high prevalence of people with asthma showed symptoms of or clinical diagnoses of anxiety or depression, which appeared to play a key role in understanding the relationship between asthma and QoL. Overall, having a diagnosis of anxiety or depression was associated with poorer QoL across all dimensions (e.g. activity limitation, physical or mental wellbeing, social or role functioning, etc.), as well as health perceptions.^24,36,46,50,54^ In particular, one study (of undergraduate students aged 18–25 years, with childhood-onset asthma) found that anxiety was significantly associated with asthma QoL, as was the interaction between anxiety and depression,^32^ while others found that generally anxiety and depression both predicted worse QoL independently (refs ^12,29,33,38,42,44,56,60^). One study found that the average asthma-related QoL scores for people with asthma and depression were 1.4 times lower compared to people with asthma and no depression.^33^ Having current depression or anxiety was associated with worse QoL than was having a lifetime diagnosis; this was in turn was greater than having no depression or anxiety.^45^ Having a history of major depression was also significantly associated with worse physical and mental functioning, compared to those with asthma and no depression.^38^ There was considerable variability across variance explained, with depression found to account for between 3%^40^ and 56%^30^ of the variance in QoL, whereas anxiety was found to account for between 2%^40^ and 68%.^21^

In contrast, one study found that having either a depressive or an anxiety disorder significantly impacted asthma QoL but having both was not significantly different than only having one,^40^ which is dissonant with other studies. Another study of 90 people with difficult asthma found that having anxiety or depression had no significant effect on QoL.^48^ In addition, although depression was associated with poorer QoL, it did not inflate the relationship between asthma severity and QoL.^29^ All other studies were significant but showed only small-to-moderate effect sizes. Having a full clinical diagnosis of anxiety or depression was not significantly worse (in terms of QoL) than having only some symptoms of anxiety and depression.

Studies also considered the impact of anxiety and depression on specific subdomains of QoL and asthma-specific QoL. Having anxiety was not associated with physical functioning, mental health or health perception,^38^ or the physical component of QoL.^20^ Depression, however, was associated with significantly poorer QoL on physical dimensions and activity limitation,^20,21,23,30,38,45,53,55,58^ although one study found significant results only for participants with uncontrolled asthma.^22^ In relation to asthma-specific QoL, depression and anxiety were significantly associated with decreased asthma-specific QoL.^17,21,23,27,28,32,33,36,37,40,50,54,55,58,61^

Nine studies looked at other mental health conditions, such as panic disorder with or without agoraphobia,^24,38,44,57^ personality disorders,^31^ alexithymia,^23^ somatization,^38^ mood disorders,^12,40,57^ schizophrenia, eating disorders, substance use disorders,^38^ and general occurrence of any psychiatric disorder.^12,17^ The results in this subtheme were mixed, but overall they suggest that the presence of an additional mental health condition is significantly associated with a decrease in QoL in patients with asthma.^12,17^ Panic disorder was also shown to be both significantly^24^ and non-significantly^57^ associated with poorer mental and physical components of QoL. Alexithymia in people with asthma was not associated with poorer QoL.^23^ Having asthma and a personality disorder was associated with lower general QoL,^31^ as well as lower scores for physical health, vitality, pain, general health, social function, mental health, and emotional role (physical function was not significant). This association was not found for people without asthma, suggesting that it is the combination of conditions (asthma and co-existing mental health conditions) that may lead to the negative impact on QoL.^31^

The emotion regulation subtheme included studies that explored the relationship between emotional states, negative affect (not related to anxiety, depression, or other mental health conditions), or coping and QoL in people with asthma. QoL in asthma was found to be influenced by affect and a predisposition to negative states, as found by four studies.^28,39,41,51^ For instance, a model of age, gender, negative affect, and medical problems accounted for 20% of symptoms and 23% of activity limitation.^39^ This was supported by findings that negative mood is associated with poor scores on both the mental and physical components of the Asthma Quality of Life Questionnaires (AQLQ),^28^ as well as a positive correlation between active coping and asthma QoL.^51^ Despite heterogeneity, the impaired QoL was associated with impulsive-careless coping^41^ and avoidant coping.^51^ Overall, the presence of psychological distress seemed to affect people with asthma more than people without asthma in terms of QoL.

Illness-related cognitions are people’s patterns of beliefs about the characteristics of their conditions, which in turn influence their appraisal of severity and can determine future behaviours.^63^ A number of illness-related cognitions and perceptions significantly predicted QoL in seven studies.^26,34,37,42,43,51,60^ For instance, asthma self-efficacy^42^ was positively associated with QoL. However, decreased QoL was significantly predicted by a series of varied illness perceptions: subjective illness severity, uncertainty in illness, illness intrusiveness,^43^ perceived disability,^60^ health beliefs and attitudes,^34^ perceived severity,^34^ level of confidence or self-efficacy in managing asthma,^51^ satisfaction with illness,^51^ anxiety sensitivity for physical concerns,^39^ and satisfaction with life.^37^ In addition, a model of subjective and objective illness severity accounted for 24% of the variance in QoL, further supporting the effect of illness perceptions on QoL.^34^

### Physical health factors

Two subthemes were generated in the physical health factors theme: additional physical conditions and BMI.

Ten papers examined additional physical conditions in relation to QoL in asthma;^25,27,34,39,46–49,52,53^ most only referred to ‘comorbidity’ or ‘medical problems’ as a measure of frequency of additional conditions.^34,36,39^ Some studies looked at both general and individual co-existing conditions^25,48,52^ and others counted chronic conditions but did not include them in further analyses.^33,36,56,59^ Of the ones that did explore individual conditions, the highest impact seemed to be provoked by musculoskeletal conditions.^25^ Similarly, statistically and clinically significant decreases in activity levels were also found for people with asthma and multimorbid conditions.^52^ Other conditions investigated included respiratory conditions,^47^ diabetes,^25,48^ obesity,^48^ hypertension,^25,39^ gastro-oesophageal reflux disorder,^48^ rhinitis,^48,49^ vocal cord dysfunction,^48^ sleep apnoea,^48^ musculoskeletal disorders,^25,39^ arthritis,^39,52^ heart disease,^25^ stroke,^39,52^ cancer,^39,52^ osteoporosis,^52^ dysfunctional breathing,^48^ headaches,^39^ and allergic status.^27,39^ The consensus was that having an additional physical condition significantly decreased QoL in asthma, the effect being amplified with the addition of further conditions.

Eleven papers exploring BMI found that it consistently influenced QoL for people with asthma both directly as a multimorbid factor and indirectly by increasing the chance of additional conditions and activity limitation.^25,26,28,29,35,42,44,45,48,56,59^ In particular, one study found that generic health status decreased for overweight and obese patients with asthma. People with asthma with obesity had on average 5.05 more restricted activity days than people without obesity or without asthma.^35^ Other studies found that increased BMI was an independent factor in predicting poorer QoL^48^ and that QoL was two times worse in overweight and three times worse in obese people with asthma.^59^ In contrast, one study found that overweight BMI made no difference; however, being obese did.^27^ Almost ½ of obese patients and 25% overweight patients had problems with mobility, pain, discomfort, self-care, and usual activities (compared to <15% people with asthma of normal weight).^26^

### Multifactorial aspects

Seven studies included statistical analyses to explore potential mechanisms for the relationship between asthma QoL and additional physical conditions, BMI, and psychological factors.^17,35,42,45,50,56,59^ Results from studies in this group are complex, indicating that people with asthma are at a higher risk of adverse outcomes (such as exacerbated symptoms or decreased QoL) if they also have a high BMI and depression.^35,42,56,59^ People with current depression and asthma are more likely to be obese and 3.9 times more likely to report fair or poor general health.^45^ A few of these studies have explored the relationship between these factors further. For example, people with asthma and obesity were more likely to have additional physical comorbidities and poorer QoL.^59^ Significant increases in major depression were associated with dyspnoea,^50^ and depression and perceived control of asthma significantly mediated between BMI and QoL.^35^ Higher BMI has also been associated with worse asthma-specific self-efficacy, which was in turn associated with decreased QoL.^42^

## Discussion

The aim of the present review was to synthesise the literature exploring health and psychological factors that influence QoL in adults with asthma. Previous evidence shows that QoL is generally lower in people with asthma and compounded by poor asthma control and severity.^13^ The narrative synthesis in the present study builds on this by identifying three themes, encompassing a number of factors that substantially explain further impairment in QoL for people with asthma. These were not limited to individual components but also combinations of co-existing conditions, risk factors, and health and psychological factors, which consistently showed a negative impact on QoL.

Anxiety and depression were the most commonly reported psychological factors associated with impaired QoL, but effects were also found for other mental health conditions, illness representations, and emotion regulation. These results are generally consistent with previous research showing not only that among people with asthma there are more people with depression than without^8^ but also with an increase in depression, the risk of asthma increased.^64^ Although the relationship between anxiety and depression and asthma-specific QoL were not further considered in the primary sources, they point towards either a link with activity limitation or a cumulative impact of the interaction between these psychological factors, which in turn affect the QoL of people with asthma. In addition, it is argued that people with asthma use more emotion-focused, and generally maladaptive, coping strategies, such as avoidance.^65^ Despite this, psychotherapy, such as cognitive-behavioural therapy and counselling has had limited effectiveness in improving asthma outcomes.^66^

Physical health factors, such as high BMI and co-occurring health conditions, were extremely common in people with asthma, consistent with existing literature.^16^ This affects QoL both directly and indirectly, affecting self-management and illness perceptions. As such, non-pharmacological treatments such as lifestyle change and activity promotion could prove effective. For instance, a higher proportion of people with asthma seem to have overweight or obese BMI^67^ and weight loss intervention studies have been associated with improvements in asthma symptoms.^68^

One of the fundamental components of reduced QoL is activity limitation, which is especially relevant to people with asthma, with or without additional conditions or psychological risk factors. This has been widely acknowledged by previous research, to the extent that it has been included as one of the components of asthma-related QoL measures, such as the AQLQ.^69^ Furthermore, it is not surprising that decreased QoL in adults with asthma is associated with depression or high BMI, both of which have been consistently associated with activity limitation (e.g. refs ^70,71^). In addition, depression was found to affect QoL on the physical components as well as the mental ones, which has interesting implications for future research and clinical practice.

It is important to note the high prevalence of anxiety, depression, and chronic conditions, despite frequent exclusion of comorbid psychiatric conditions. This was found throughout the included papers and is consistent with previous research (e.g. refs ^8,16^). This does not only mean that psychological and health factors significantly add to the burden of living with asthma but also that the occurrence of psychological dysfunction and health risk factors seem to be common in people with asthma. In addition, the complex nature of patients with chronic diseases such as asthma, with factors interacting, adds to the negative experience of living with asthma. Results are similar to previous meta-analyses and reviews,^8,72^ pointing towards conclusive evidence that additional factors (physical or psychological) decrease QoL and functionality in asthma. Finally, these effects were consistent, regardless of the measure of QoL used (asthma specific, health related, or general). This suggests that the identified factors may affect people with asthma more than people without asthma or that the cumulative impact of comorbidities is greater than arithmetically assumed.

The quality of the present review needs to be discussed in relation to the methodology and robustness of the synthesis, determined by the quantity and quality of individual studies included.^73^ The quality assessment identified that most studies were of a reasonable quality overall, although all papers had one or two elements that were of a slightly lower quality (this included aspects such as recruitment from only one hospital reducing generalizability or self-report vs objective measurement of weight for BMI calculations). However, this was not problematic for the purposes of this review as the focus was to identify potential factors considered in research rather than classify the methodological quality used to measure their impact on QoL. In addition, the search terms in this review could have limited the number and kind of studies included. For instance, not every potential comorbid condition was listed. This could be a focus for future research. Socio-demographic factors were not included, which can be considered a limitation; however, the breadth of the area was deemed too much for the scope of the present review and could also be the focus of future research. The majority of included studies were observational and as such could not be used to determine causal mechanisms. However, the aim of this review was only to identify potential factors involved in decreased QoL in asthma, rather than build a causal model. Similarly, the impact of individual factors was not measured and could be explored in future research.

A strength of the present review is that it uses a novel approach to QoL in asthma, by systematically taking into account additional aspects that influence the experience of living with asthma and impact QoL. Results suggest both a direct association of the identified aspects, as well as indirectly through interactions with other aspects of living with asthma, such as overarching illness perceptions and activity limitation. The present review emphasizes some interesting and novel findings for asthma and QoL research. Three main implications for future research and practice are proposed. First, for future research, the findings of this review should be used to further explore and understand the factors impacting QoL in people with asthma. It is crucial to explore the needs and experience of patients with complex medical problems, in order to unpick the different factors impacting on QoL. Second, the results are relevant for practitioners, particularly in primary care, as they draw attention to the prevalence of various physical and mental health factors that can interact and affect asthma outcomes. This could influence training or guidelines on potential factors to consider during appointments and consultations. Finally, most current non-pharmacological interventions for patients with chronic conditions tend to overlook the complex needs of patients in a multimorbidity context. As such, it is suggested that future intervention development should use a personalized, tailored approach that aims to address the needs of patients with complex medical problems in the wider context of their experience of living with asthma.

This review demonstrates that the themes and factors identified through inductive narrative synthesis illustrate that QoL in asthma cannot be determined in a simplistic way. The findings suggest a complex experience in living with asthma, one that has a stronger impact on QoL than the sum its of parts. People with asthma and their QoL cannot be viewed separately from the psychological and other health elements that they experience. Future research is encouraged to take a function-oriented approach to QoL in asthma, including management of multimorbid conditions when planning studies; clinical practice should also acknowledge the additional and complex needs of people with asthma by offering relevant, person-based tailored interventions.

## Method

### Search strategy

The initial search was carried out in April 2017 and was updated in January 2019. Databases searched included MEDLINE, EMBASE, PsycINFO, the Cochrane Library, and Web of Science. Search terms used comprised a combination of the following key terms: asthma (MESH term), psychological/psychosocial and factor/determinant/predictor, comorbid, multimorbid, anxiety, depression, illness perception, illness cognition, illness representation, locus of control, self-efficacy, risk factor, quality of life, health-related quality of life, wellbeing, distress, health status, burden. In addition, a hand search of all the references of included papers was performed as well as a grey literature search on Google Scholar.

### Study selection

Studies were included if they investigated psychological or physical health factors and included QoL in adults with asthma as primary or secondary outcome. Psychological factors were considered any modifiable factors, including thoughts, beliefs, attitudes, or emotions of people with asthma, as well as the presence of any co-occurring mental health condition. Physical health factors were defined as any physical comorbid or multimorbid condition or risk factor. These were chosen to allow as much inclusivity as possible and to reflect the exploratory nature of this review. Intervention studies were excluded, as they rarely considered the impact of health or psychological factors on QoL but rather investigated how interventions improved asthma outcomes. Studies were excluded if they were conference abstracts, reviews, or not primary research or the full text not in English, German, or Spanish language.

### Data extraction and quality appraisal

Data extracted comprised authors, year of publication, study sample, predictors, QoL measurement (outcome), and findings. The AXIS tool^74^ was used to assess the quality of included papers. This contains questions on study design, sample size justification, target population, sampling frame, sample selection, measurement validity and reliability, and overall methods and does not offer a numerical scale. No papers were excluded or weighted based on the quality assessment.

### Data synthesis

Owing to heterogeneity of QoL measures and the range of variables used in the included studies, narrative synthesis was used to describe and group similar findings, explore patterns identified in the literature, and develop a narrative account of the results.^73^ This is an approach to systematic reviews involving the synthesis of findings from multiple sources and relies primarily on word and text to summarise the findings.

All data generated or analysed during this study are included in this published article.

### Reporting summary

Further information on research design is available in the Nature Research Reporting Summary linked to this article.

## Supplementary information

Reporting Summary
